# Response of serum biochemical profile, antioxidant enzymes, and gut microbiota to dietary Hong-bailanshen supplementation in horses

**DOI:** 10.3389/fmicb.2024.1327210

**Published:** 2024-02-20

**Authors:** Jinxue Ding, Bolin Gu, Jinwu Meng, Mengxin Hu, Wenjia Wang, Jiaguo Liu

**Affiliations:** Institute of Traditional Chinese Veterinary Medicine, College of Veterinary Medicine, Nanjing Agricultural University, Nanjing, China

**Keywords:** HBLS, horse, biochemical profile, antioxidant enzymes, gut microbiota

## Abstract

**Background:**

Traditional Chinese medicine (TCM) is widely used in humans and animals, which is very important for health. TCM affects the body ‘s immunity and changes in intestinal flora. This study was conducted to investigate the effects of dietary Hong-bailanshen (HBLS) supplementation in horses on serum biochemical profile, antioxidant enzymes and gut microbiota.

**Methods:**

In this study, five horses were selected. On day 0, 14, 28, blood samples and feces were collected on days 0, 14, and 28 to analyse gut microbiota, serum biochemical and redox indexes.

**Results:**

The results showed that the addition of HBLS to horse diets significantly decreased the level of alanine aminotransferase, alkaline phosphatase, creatine kinase and malondialdehyde (*p* < 0.05, *p* < 0.01) and significantly increased the activity of total antioxidant capacity, superoxide dismutase and catalase (*p * < 0.05, *p * < 0.01). Compared with day 14, the levels of alanine aminotransferase, alkaline phosphatase and creatine kinase were significantly decreased; however, the level of catalase was significantly increased in the horses continuously fed with HBLS for 28 days (*p * < 0.05, *p* < 0.01). Alpha diversity analysis was performed that chao1 (*p * < 0.05), observed_specicies, faith’pd and goods_coverage upregulated in the horses fed HBLS. A total of 24 differential genera were detected adding HBLS to diet increased the abundance of *Bacillus, Lactobacillaceae, Leuconostocaceae, Christensenellaceae, Peptostreptococcaceae, Faecalibacterium, Erysipelotrichaceae, Pyramidobacter, Sphaerochaeta, WCHB1-25, Bacteria, Oscillospira,* and *Acetobacteraceae*, while reduced *Aerococcus, EtOH8, Syntrophomonas, Caulobacter, Bradyrhizobiaceae, W22, Succinivibrionaceae,* and *Desulfovibrio* (*p* < 0.05, *p* < 0.01).

**Conclusion:**

Adding HBLS to the diet could be a potentially effective strategy to improve horses’ health.

## 1 Introduction

Horses have played a vital role in human development and expansion of human settlements (Wutke et al., 2016). “The leisure equestrian performance, the athletics competition and the equestrian teaching and the experience” for the present sports horse industry is growing in China. Many scholars have researched the athletic ability and disease prevention of sports horses (Clayton, 2016; Arnold et al., 2020; Ursini et al., 2022). At the same time, they pay close attention to the welfare of sports horses and strictly control the use of antibiotics in sports horses (McLean and McGreevy, 2010). TCM plays a vital role in animal nutrition, disease prevention, and treatment with the characteristics of pollution-free, no residue, and green environmental protection (Gao and Tong, 2006). Therefore, TCM additives have become the focus of attention and research among equestrian industry experts.

Chinese herbs have been utilized in medicine for thousands of years in China (Lo and Shaw, 2019). In some Eastern European countries, herbal plants are trendy and can be obtained for free from nature without extra costs to feed producers (Vlaicu et al., 2022). Therefore, they can be considered one of the first functional food ingredients. *Rhodiola rosea*, Atractylodes, Gei Herba, and Codonopsis are commonly used traditional Chinese herbs with various health benefits (Hung et al., 2011; Ishaque et al., 2012; Gao et al., 2018; Zhu et al., 2018; Kashchenko et al., 2023). Numerous researchers have recognized their safety, and these herbs are widely used in animals (Zhao et al., 2006; Gupta et al., 2008; Ding et al., 2023). These herbs contain several active components that have been shown to benefit animals, such as flavone, polysaccharides, and polyphenols (Kosakowska et al., 2018; Luan et al., 2021; Zhou et al., 2022). *Rhodiola rosea* has been studied by many scholars for its anti-oxidation and improvement of intestinal flora (Zhou et al., 2014; Olennikov et al., 2020). Gei Herba can improve hematopoietic function (Zhao et al., 2020). Atractylodes and Codonopsis have the functions of protecting the liver, regulating immunity, and improving the gastrointestinal tract (Ming et al., 2017; Qu et al., 2022). An herbal blend made up of many different plants has several active components that could be more biologically effective than a single herbal extract (Yuan et al., 2022; Zheng et al., 2023).

Intestinal flora is an essential factor in maintaining the health condition of the host (Kakakhel et al., 2023a). Important functions such as absorbing nutrition, synthesizing short-chain fatty acids, and inhibiting the colonization of other pathogenic bacteria are performed by the intestinal flora in the hindgut of a horse (Arnold et al., 2021; Parker et al., 2024). Intestinal bacteria may belong to several functional groups; for example, Lactobacillus spp. can hydrolyze starch and produce lactic acid as well. These functional groups are vital to a horse’s plant-rich diet (Julliand et al., 1999; Harlow et al., 2016). Intestinal flora also plays an important role in the internal transformation of Chinese herbs (Li et al., 2021), and it has become a popular subject in life science research.

This study selected the Chinese herbal medicines *Rhodiola rosea*, Atractylodes, Gei Herba, and Codonopsis and formulated HBLS according to the theory of “replenishing qi and strengthening the spleen” of TCM. Equestrian horses in the racecourse were used as test animals to explore the response of serum biochemical profile, antioxidant enzymes, and microbial community to dietary HBLS in sports horses. The design of this study has been thoroughly informed by the principles of Chinese medicine theory and comprehensively assessed through a multifaceted array of indicators. The primary objective is to establish a scientific foundation for the application of Chinese medicine to enhance the overall health of sports horses.

## 2 Materials and methods

### 2.1 Preparation of HBLS

HBLS was composed of four Chinese herbs, including *Rhodiola rosea* (*Rhodiola rosea L*), Atractylodes (*Atractylodes macrocephala Koidz.*), Gei Herba (*Geum aleppicum Jacq.*), and Codonopsis (*Codonopsis pilosula (Franch.) Nannf.*), which were purchased from Beijing Tongrentang Medicine Company (Nanjing, China). The four kinds of TCM were stewed for 1 h. After the liquid had been filtered, the herb residue was reintroduced to the water and brought to a second boil before being blended with the first liquid and strained again. The liquid was then lyophilized, and auxiliary ingredients were added to create powder (1: 1 power to raw herb ratio).

### 2.2 Animal experiment design

Five well-trained and healthy horses, 10 ± 1 years old and 400 ± 15 kg, were selected from the Flying Marode International Equestrian Institute (Nanjing, China). They received the same diet, usually offered two times a day. In the morning, they each received 2 kg of commercial feed (Luxury horse complete formula feed, China) and 2 kg of alfalfa hay. In the evening, the horses were each fed 2 kg of commercial feed, 2 kg of alfalfa hay, and 100 g of HBLS. The HBLS was added for 28 days. Water was provided *ad libitum*.

**Figure 1:**
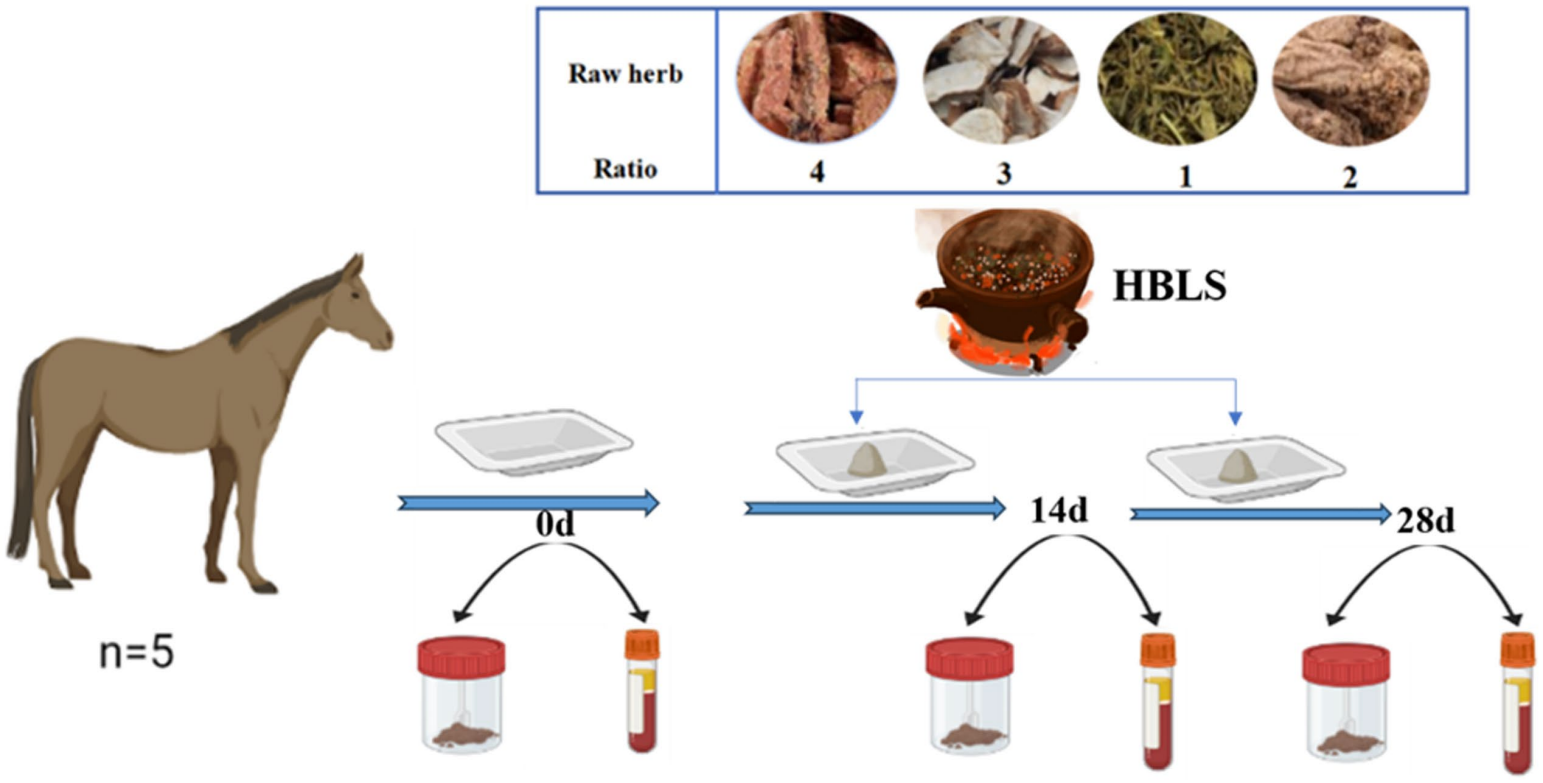
Study design for the experiment.

Samples were taken on day 0, day 14, and day 28, respectively (Figure 1).

### 2.3 Determination of serum biochemical profile and antioxidant enzymes

The blood samples of horses were centrifuged at 3500 rpm for 10 min, and the supernatant was divided into 1.5 mL Eppendorf tubes and stored at −20°C for future analysis.

The serum biochemical profile of horses was determined using an automatic biochemical analyzer (Alovet Li200, ALOVET Co., Ltd., China). Biochemical index-related kits were purchased from Alovet Co., Ltd., and the detection methods were strictly in accordance with the requirements of the kit. Parameters determined were aspartate aminotransferase (AST), alanine aminotransferase (ALT), alkaline phosphatase (ALP), creatine kinase (CK), lactate dehydrogenase (LDH), gamma-glutamyl transferase (GGT), glucose (GLU), triglyceride (TG), total cholesterol (CHOL), total protein (TP), and albumin (ALB).

The oxidative stress was estimated in horses by detecting the malondialdehyde (MDA) level (S0131S, Beyotime, Shanghai, China), superoxide dismutase (SOD) (A001-3-2, Nanjing Jiancheng, Nanjing, China), catalase (CAT) (BC0205, Solarbio, Beijing, China) activities, and total antioxidant capacity (T-AOC) (BC1312, Solarbio, Beijing, China) following the protocol of the detection kits.

### 2.4 DNA extraction and sequencing

Microbial DNA was extracted from horse feces (*N* = 5) by adding HBLS on day 0, day 14, and day 28, respectively, using the rapid DNA fecal mini kit from Qiagen (Germany) according to the manufacturer’s instructions. DNA was quantified by NanoDrop (Thermo Scientific, United States), and the quality of DNA was detected by 1.2% agarose gel electrophoresis. The V3/V4 regions of the 16S rRNA gene of bacteria were amplified using primer pairs of 338F (ACTCCTACGGGAGGCAGCAG) and 806R (GGACTACHVGGGTWTCTAAT) as reported in previous studies (Hu et al., 2016; Wang et al., 2019). Then, magnetic beads (Vazyme VAHTSTM DNA Clean Beads) with a volume of 0.8 times were added to 25 μL of PCR product for purification and quantified using the Microplate reader (BioTek, FLx800). Finally, according to the instructions, it was followed by sequencing via the Illumina MiSeq platform (Bioyi Biotechnology Co., Ltd., China).

### 2.5 Gut microbiota analysis

First, the original off-machine data of high-throughput sequencing were preliminarily screened according to the sequence quality, and sequence denoising or OTU clustering was performed according to the analysis process of Qiime2 DADA2 (Callahan et al., 2016) or Vsearch software (Rognes et al., 2016). The alpha diversity of the intestinal bacterial community, including Chao1, Simpson, Shannon, Pielou’s evenness, observed species, Faith’s PD, and Goods coverage, was analyzed using Qiime2. Then, at the ASV/OTU level, the distance matrix of each sample was calculated, and the difference and significance of beta diversity among different samples (groups) were measured by various unsupervised sorting and clustering methods combined with corresponding statistical test methods. Second, at the level of taxonomic composition, the differences in species abundance among different samples (groups) were further measured by various non-supervised and supervised sorting, clustering, and modeling methods combined with corresponding statistical test methods, attempting to find the signature species. According to the species distribution in each sample, the association network is constructed, the topological index is calculated, and the key species are tried to be identified. Finally, based on the results of 16S rRNA gene sequencing, the metabolic function of the samples can be predicted, the differential pathway can be found, and the species composition of the specific pathway can be obtained.

### 2.6 Statistical analysis

Statistical analyses were conducted with a one-way ANOVA followed by LSD’s multiple comparisons tests using SPSS version 26 software (SPSS 26, IBM, American) and GraphPad Prism 8 software (GraphPad Prism 8.4.2 software, Inc., San Diego, CA). Data are presented as mean ± standard deviation (SD). *p < 0.05 and **p < 0.01 were considered statistically significant.

## 3 Results

### 3.1 The effect of HBLS on serum biochemical profile

**Figure 2:**
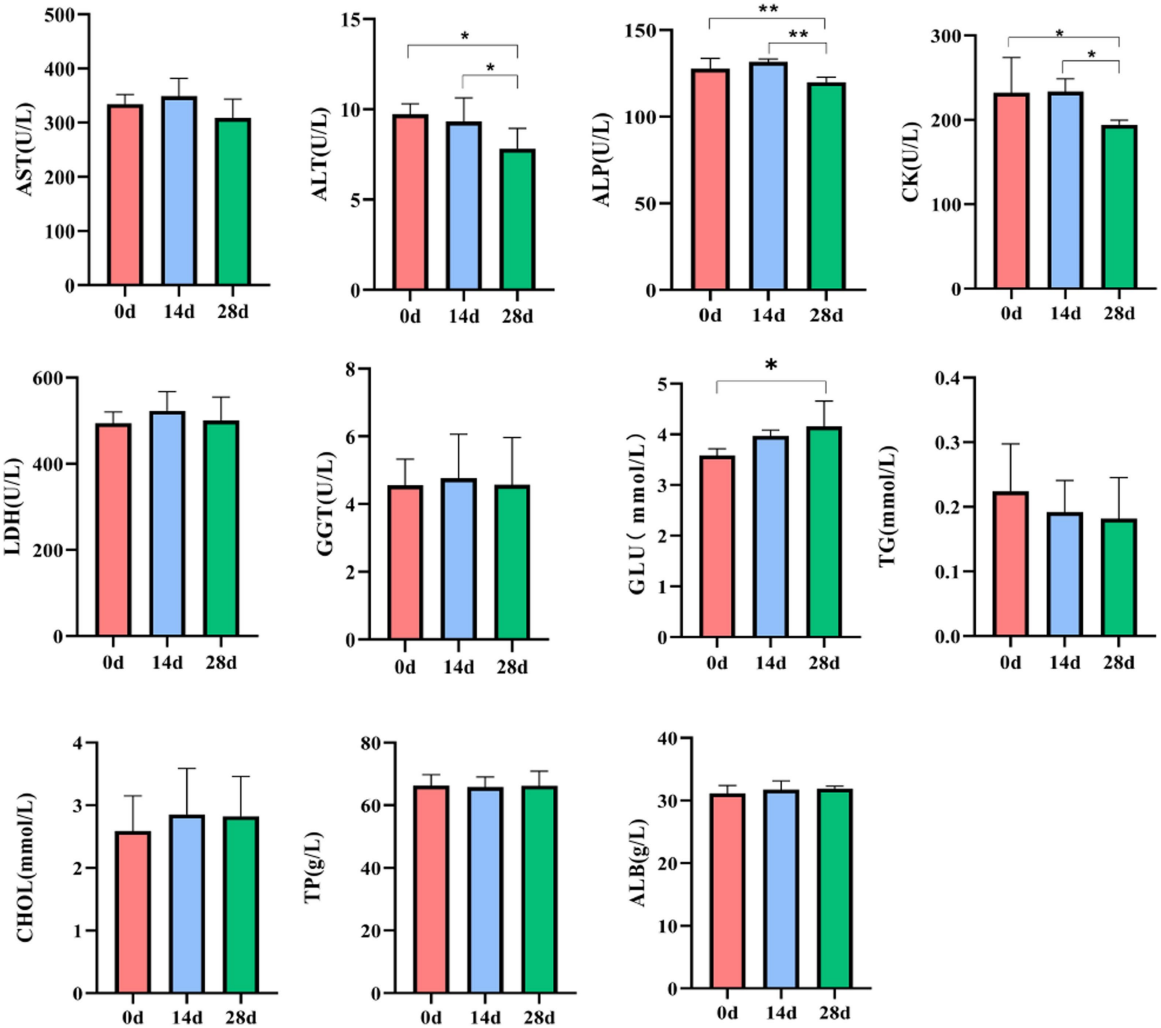
Effect of HBLS on the serum biochemical profile.

In horses’ serum, no noticeable difference was found in LDH levels on day 0, day 14, and day 28. Compared to day 0, the level of ALT, ALP, and CK of horses was significantly decreased (*p* < 0.05, *p* < 0.01); however, the level of GLU of horses fed with HBLS for 28 days was significantly increased (*p* < 0.05). Compared to day 14, the level of ALP and CK in horses fed with HBLS for 28 days was significantly decreased (*p* < 0.05, *p* < 0.01) (Figure 2).

### 3.2 The effect of HBLS on antioxidant enzymes

**Figure 3:**
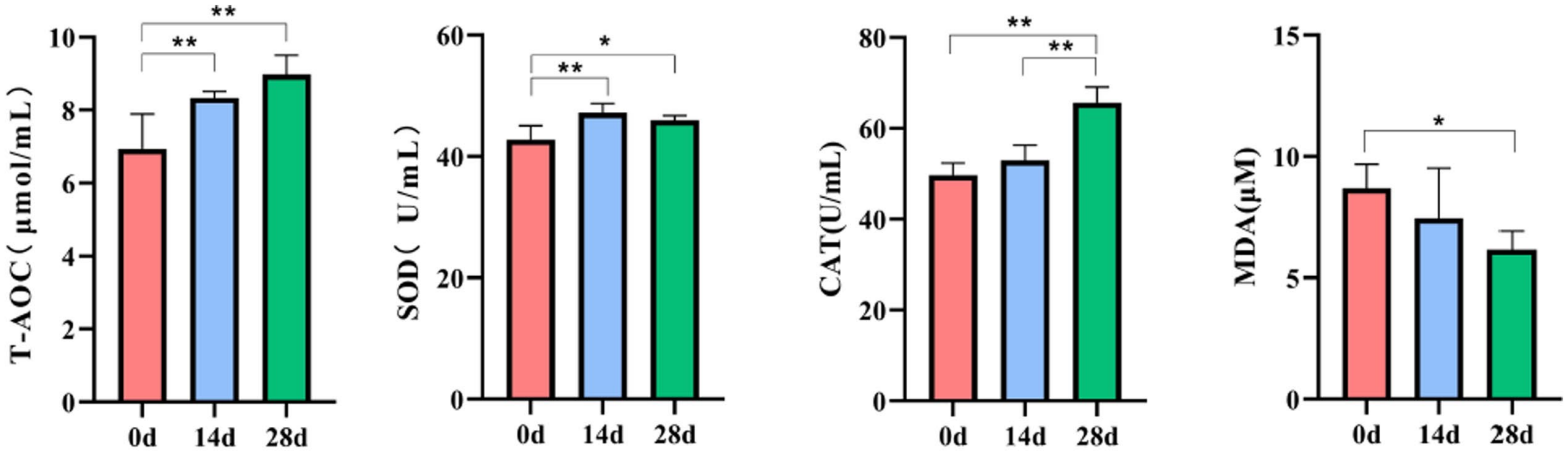
Effect of HBLS on antioxidant enzymes.

The antioxidant indices were determined according to T-AOC, SOD, CAT, and MDA kits. Significantly increased activities of T-AOC and SOD were observed on day 0 compared to day 14 (*p*<0.01). While on day 28, the activities of T-AOC, SOD, and CAT were significantly increased (*p*<0.01), and the activities of MDA were decreased (*p*<0.05) compared to day 0. A significant increase in CAT was also observed between days 14 and 28 (*p*<0.01) (Figure 3).

### 3.3 The structure and diversity of horses’ gut microbiota

**Figure 4:**
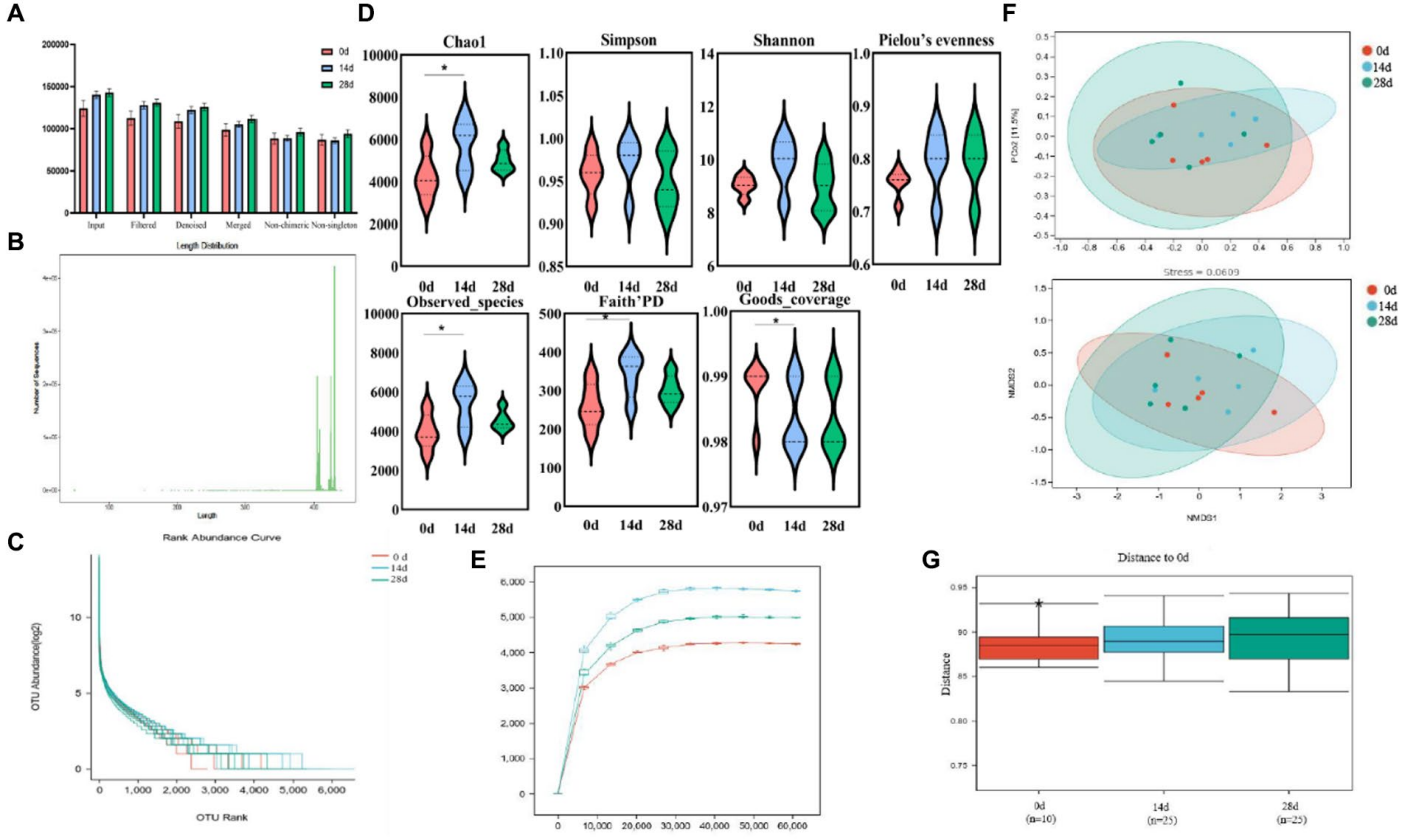
HBLS changed the structure and diversity of the gut microbiota of horses. **(A)** Sequencing data statistical analysis, **(B)** length distribution of sequencing data, **(C)** rank abundance curve **(D)** alpha diversity index analysis, **(E)** sample rarefaction curves, **(F)** beta diversity analysis, and **(G)** group difference analysis. Significance is presented as ^*^p < 0.05, ^**^p < 0.01. Data are presented as the mean ± SEM (*n* = 5).

The current study achieved over 92,000, 84,000, 80,000, 72,000, 65,000, and 64,000 input, filtered, denoised, merged, non-chimeric, and non-singleton data in different horse samples (Table 1). No noticeable difference was found in the sequencing data on day 0, day 14, and day 28 (Figure 4A). Most sequence lengths were approximately 430 bp (Figure 4B). Flat broken lines were present in all samples, reflecting the evenness and richness of the OTU composition (Figure 4C). Meanwhile, the multi-sample rarefaction of all samples showed sufficient species coverage (Figure 4E). The alpha diversity index values of Chao1, Simpson, Shannon, Pielou’s evenness, observed species, Faith’s PD, and Goods coverage are shown in Table 2. Alpha diversity analysis was conducted by examining the diversity indices, which revealed that on day 14, the values of chao1 (*p* < 0.05), observed_species (*p* < 0.05), Faith’s PD (*p* < 0.05), and goods_coverage (*p* < 0.05) indices in the horses fed HBLS were higher than those not fed HBLS (Figure 4D). Beta diversity analysis indicated that adding HBLS to the diet could change the intestinal colonies of horses (Figure 4F). UPGMA analysis found that the branch length without adding HBLS to the diet was relatively shorter than adding HBLS to the diet of horses (Figure 4G).

**Table 1:** Statistical analysis of sample sequencing data.

	Sample ID	Input	Filtered	Denoised	Merged	Non-chimeric	Non-singleton
0d	CG1	140,239	126,201	122,546	111,856	98,843	97,578
	CG2	92,614	84,204	80,859	72,159	65,463	64,137
	CG3	116,865	106,841	102,626	91,747	82,344	80,899
	CG4	126,205	113,835	111,382	105,879	98,245	97,697
	CG5	145,231	132,049	126,783	111,270	96,692	94,219
14d	EG1_1	138,797	126,831	122,848	110,461	98,550	97,233
	EG1_2	137,931	126,334	120,106	99,527	81,221	78,277
	EG1_3	130,399	117,036	112,042	97,847	82,461	80,531
	EG1_4	157,865	144,136	136,695	116,116	92,417	88,517
	EG1_5	136,245	125,249	119,252	101,957	88,189	85,603
28d	EG2_1	152,939	140,271	135,321	120,423	103,471	101,562
	EG2_2	143,985	131,368	127,284	115,020	100,067	98,466
	EG2_3	151,366	138,948	134,035	120,072	105,733	104,109
	EG2_4	129,568	118,930	114,227	100,752	83,467	81,286
	EG2_5	135,316	124,453	118,196	101,024	87,413	84,907

**Table 2:** Statistical analysis of the alpha diversity index.

	Sample	Chao1	Simpson	Shannon	Pielou’s evenness	Observed_species	Faith’PD	Goods_coverage
0 d	CG1	4056.96	0.969985	9.0359	0.762497	3692.6	237.005	0.988191
	CG2	3765.53	0.924421	8.46757	0.714465	3695.7	246.125	0.994484
	CG3	4637.35	0.954183	9.23868	0.763629	4385.1	291.499	0.989002
	CG4	3003.15	0.987904	8.91992	0.778985	2,799	185.97	0.993432
	CG5	5784.35	0.955868	9.45077	0.764637	5256.2	341.49	0.981937
14 d	EG1_1	4177.13	0.924708	8.32001	0.699359	3812.8	253.329	0.98775
	EG1_2	6330.43	0.991958	10.4254	0.830666	5999.8	377.11	0.983494
	EG1_3	4888.63	0.982841	9.6203	0.790257	4619.9	313.927	0.987743
	EG1_4	7116.74	0.996658	10.9009	0.859487	6576.4	397.706	0.977942
	EG1_5	6186.02	0.973454	10.0367	0.803253	5773.1	363.004	0.982785
28 d	EG2_1	4857.87	0.982999	9.51474	0.787182	4350.9	282.856	0.984121
	EG2_2	4513.81	0.90945	8.07955	0.674106	4055.3	257.059	0.985459
	EG2_3	5237.97	0.99242	10.1481	0.831668	4712.1	320.211	0.984292
	EG2_4	4564.27	0.92727	8.07793	0.669528	4285.1	291.328	0.986975
	EG2_5	5737.32	0.940127	9.03026	0.729073	5352.3	356.239	0.98345

### 3.4 The taxon composition of horses’ gut microbiota

**Figure 5:**
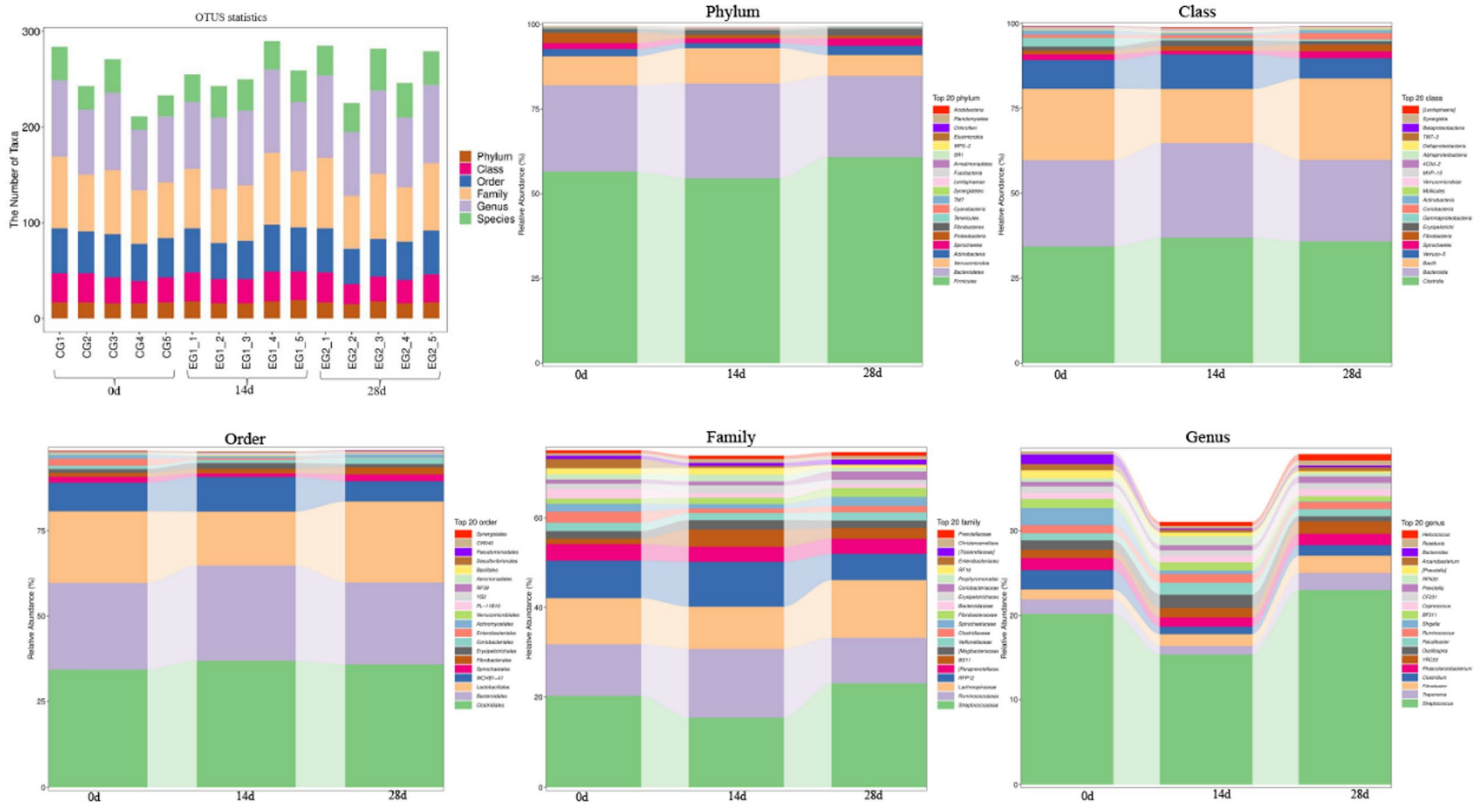
Effect of dietary HBLS on the relative abundance of gut microbiota in different taxa levels.

The number of taxa contained in different levels of phylum, class, order, family, genus, and species is shown in Figure 5. At the phyla level, the dominant phyla of horses fed with HBLS for 0, 14, and 28 days were *Firmicutes, Bacteroidetes,* and *Verrucomicrobia*. At the class level, the dominant class of horses fed with HBLS for 0, 14, and 28 days were *Clostridia, Bacteroidia,* and *Bacilli*. At the order level, the main order of horses fed with HBLS for 0, 14, and 28 days was *Clostridiales, Bacteroidales,* and *Lactobacillales*. At the family level, the main family of horses fed with HBLS for 28 days was *Streptococcaceae* (23.06%), *Bacteroidales* (14.48%), and *Lachnospiraceae* (12.28%), while the horses fed with HBLS for 0 and 14 days were *Streptococcaceae, Bacteroidales,* and *Ruminococcaceae*. At the genus level, the main genera of horses fed with HBLS for 28 days were *Streptococcus* (23.02%), *Bacteroidales* (14.49%), and *Lachnospiraceae* (8.17%), while the horses fed with HBLS for 0–14 days were *Streptococcus, Bacteroidales,* and *Ruminococcaceae*.

**Figure 6:**
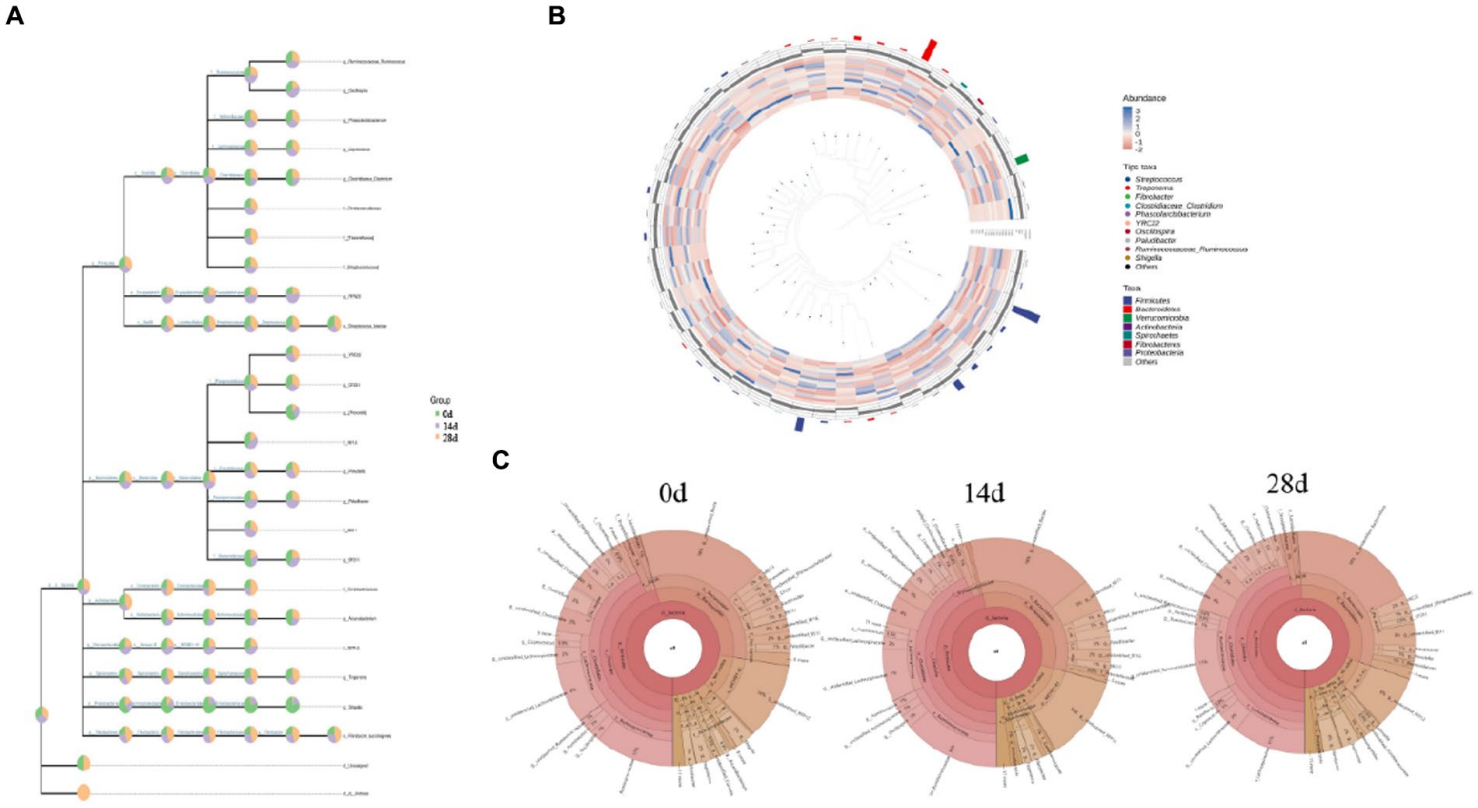
Species composition analysis of gut microbiota of horses. **(A)** Classification levels tree diagram, **(B)** GraPhlAn evolutionary tree diagram, and **(C)** Krona species composition diagram.

The classification level tree diagram showed that the proportion of *Prevotella, BF311, Arcanobacterium, Treponema,* and *Shigella* was higher in horses fed without HBLS; the proportion of *Oscillospira, RFN20, RF16, Paludibacter, BS11,* and *BF11.* The classification levels tree diagram showed that the proportion of *Prevotella*, *BF311, Arcanobacterium, Treponema,* and *Shigella* in horses fed with HBLS for 14 days was relatively high; the proportion of *Coprococcus, Tissierellaceae, Luteciae, YRC22, Prevotella, Treponema,* and *Succinogenes* in horses fed with HBLS for 28 days was relatively high (Figure 6A). GraPhlAn evolutionary tree diagram showed that the abundance of *Streptococcus, Treponema, Fibrobacter, Clostridiaceae Clostridium, Phascolarctobacterium, YRC22, Oscillospira, Paludibacter, Ruminococcaceae Ruminococcus*, and *Shigella* depicted in various colors were found to be significantly different among different horse groups (Figure 6B). Krona species composition diagram indicated that the main genera were *Bacteroidales* (18%), *Ruminococcaceae* (11%), and *RFP12* (10%) of horses fed without HBLS, *Bacteroidales* (18%), *Ruminococcaceae* (14%), and *RFP12*(12%) of horses fed with HBLS for 14 days, and *Bacteroidales* (19%), *Ruminococcaceae* (11%), and *Lachnospiraceae* (11%) of horses fed with HBLS for 28 days (Figure 6C).

**Figure 7:**
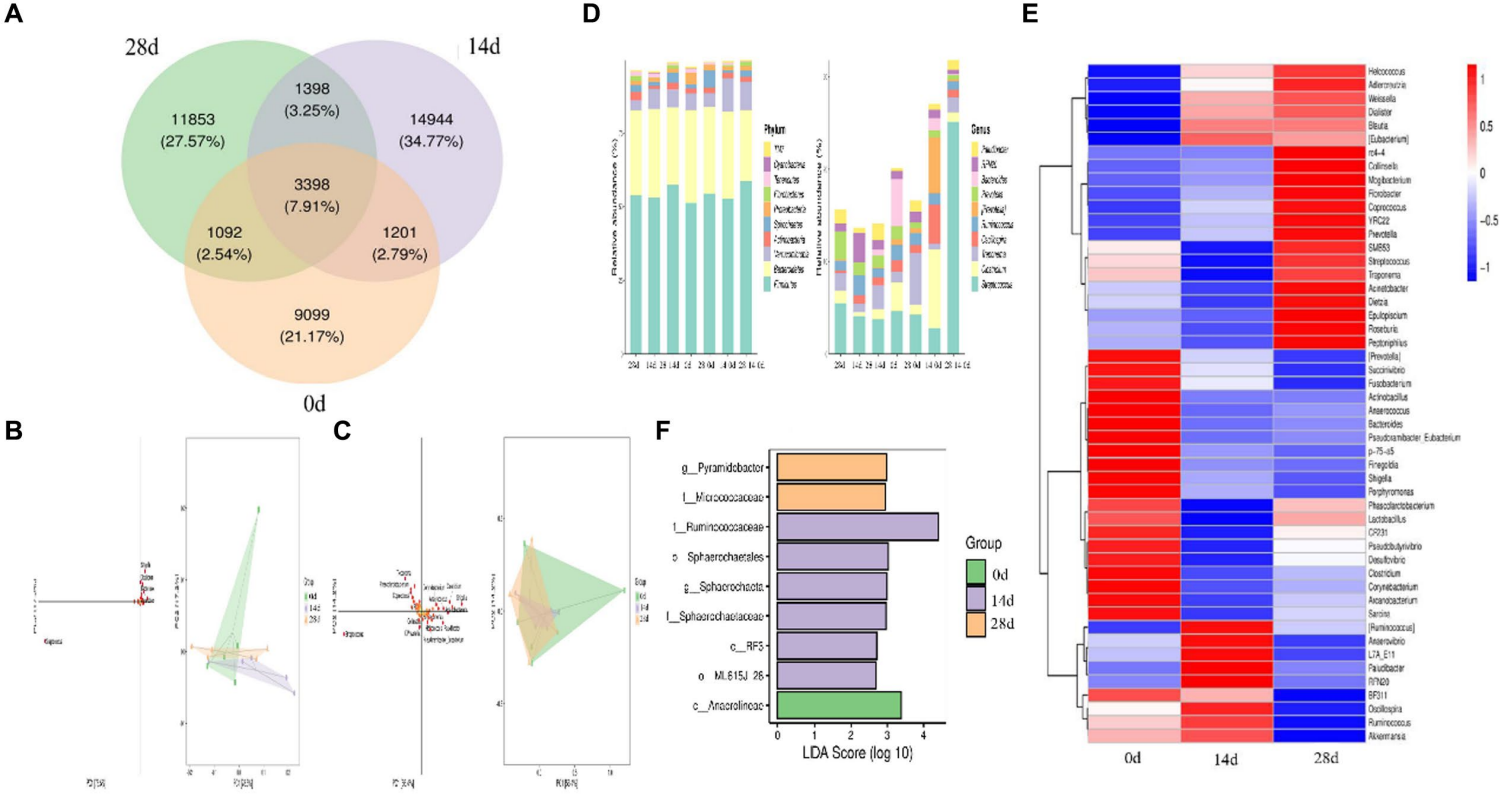
Different species and marker species analysis in horses. **(A)** Venn diagram, **(B)** PCA, **(C)** OPLS-DA, **(D)** bar chart of ASV/OTU numbers in different regions of the Venn diagram, **(E)** genera composition heatmap, and **(F)** LEfSe analysis.

In the study, we conducted Venn diagram analysis, created bar charts of ASV/OTU numbers in different regions of the Venn diagram, bar graphs of ASV/OTU abundance in different regions of the Venn diagram, and genera composition heatmap, and conducted PCA and OPLS-DA analysis. The results showed that a total of 3,398 (7.91%) shared ASVs were found among all different times of horses, 4,599 (10.77%) ASVs were shared on day 0 and day 14, 4,490 (10.45%) ASVs were shared on day 0 and day 28, 4,796 (11.16%) ASVs were shared on day 14 and day 28, 9,099 (21.17) ASVs were independent on day 0, 14,944 (34.77%) ASVs were independent on day 14, and 11,853 (27.57%) ASVs were independent on day 28 (Figure 7A). Then ASV/OTU abundance was explored in different regions of the Venn diagram. The results showed that at the phylum level, the intestinal flora of day 0, day 14, and day 28 shared *Firmicutes, Bacteroidetes, Verrucomicrobia, Actinobacteria, Spirochaetes, Proteobacteria, Fibrobacteres, Tenericutes,* and *TM7.* At the genus level, the intestinal flora of day 0, day 14, and day 28 shared *Streptococcus, Clostridium, Treponema, Oscillospira, Ruminococcus, Prevotella, Bacteroides, RFN20,* and *Paludibacter* (Figure 7D). PCA analysis showed that *Shigella, Clostridium, Bacteroides, Arcanobacterium,* and *Streptococcus* were the leading general. The distance between points of day 0 projected on the coordinate axis was farther than day 14 and day 28, which revealed a difference between day 0, and day 14 and day 28, respectively (Figure 7B). Additionally, the results of the OPLS-DA study matched those of the PCA analysis (Figure 7C). It is depicted in the heatmap that *Helcococcus, Adlercreutzia, Weissella, Dialister, Blautia,* and *Eubacterium* are shown in blue color in 0d rather than in 14d and 28d; *Prevotella, Succinivibrio, Fusobacterium, Actinobacillus, Anaerococcus, Bacteroides, Pseudoramibacter, p-75-a5, Finegoldia, Shigella, Porphyromonas, CF231, Pseudobutyrivibrio, Desulfovibrio, Clostridium, Corynebacterium, Arcanobacterium,* and *Sarcina* are shown in red color on day 0 rather than on days 14 and 28 (Figure 7E). Using LEfSe analysis, biomarker bacteria in horses were discovered, which were from the class *Anaerolineae* on day 0, family *Ruminococcaceae,* order *Sphaerochaetales*, genus *Sphaerochaeta*, family *Sphaerochaetaceae*, class *RF3*, and order *MLJ-28* on day 14, genus *Pyramidobacter*, and family *Micrococcaceae* on day 28 (Figure 7F).

**Figure 8:**
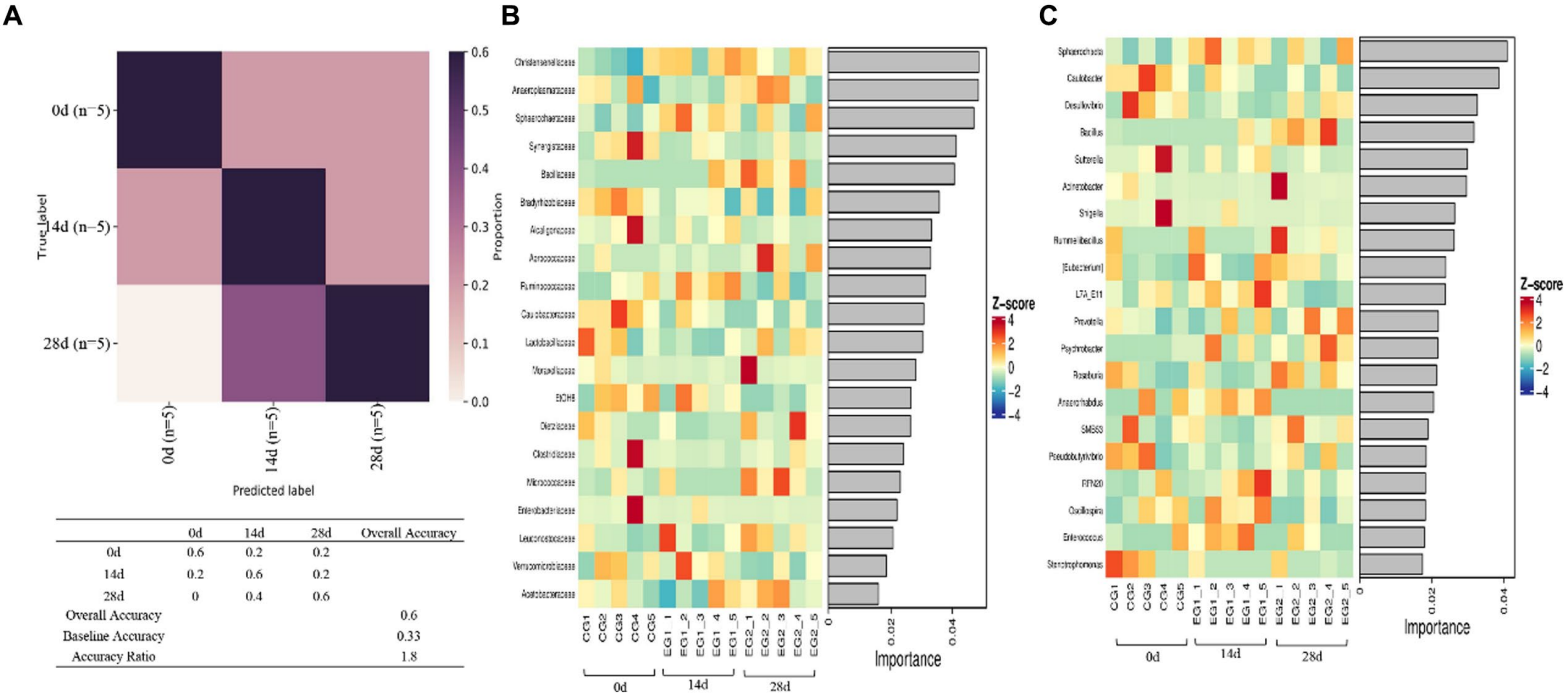
Random forests. **(A)** Model accuracy, **(B)** family, and **(C)** genus.

Random forest analysis was conducted with an accuracy ratio of 1.8, with overall accuracy and baseline accuracy of 0.6 and 0.33, respectively (Figure 8A). Important families among horses are shown in the results, including *Christensenellaceae, Anaeroplasmataceae, Sphareochaetaceae, Synergistaceae,* and *Bacillaceae* (Figure 8B); important genera among horses are shown in the results, including *Sphareochaeta, Caulobacter, Desulfovibrio, Bacillus,* and *Sutterella* (Figure 8C).

A metastats analysis was conducted at the genera level to explore further the impact of HBLS on the makeup of gut microbes; 24 genera were detected in horses. The abundance of *Christensenellaceae* (*p* < 0.05), *Sphaerochaeta* (*p* < 0.05), *WCHB1-25* (*p* < 0.05), and *Bacteria* (*p* < 0.05) in 14d was significantly higher than in 0d, respectively. While *Aerococcus* (*p* < 0.05), *Syntrophomonas* (*p* < 0.05), *Bradyrhizobiaceae* (*p* < 0.05), *Acetobacteraceae* (*p* < 0.01), *W22* (*p* < 0.05), and *Succinivibrionaceae* (*p* < 0.05) in 14d were lower on day 0, respectively. Between days 0 and 28, a significant increase was observed in the abundance of *Bacillus* (*p* < 0.05), *Lactobacillaceae* (*p* < 0.05), *Leuconostocaceae* (*p* < 0.05), *Peptostreptococcaceae* (*p* < 0.05), *Faecalibacterium* (*p* < 0.05), *Erysipelotrichaceae* (*p* < 0.05), and *Pyramidobacter* (*p* < 0.05). The abundance of *Aerococcus* (*p* < 0.05), *EtOHS* (*p* < 0.05), *Syntrophomonas* (*p* < 0.05), *Caulobacter* (*p* < 0.05), *Bradyrhizobiaceae* (*p* < 0.05), *Acetobacteraceae* (*p* < 0.05), and *W22* (*p* < 0.05) has dropped significantly.

**Figure 9:**
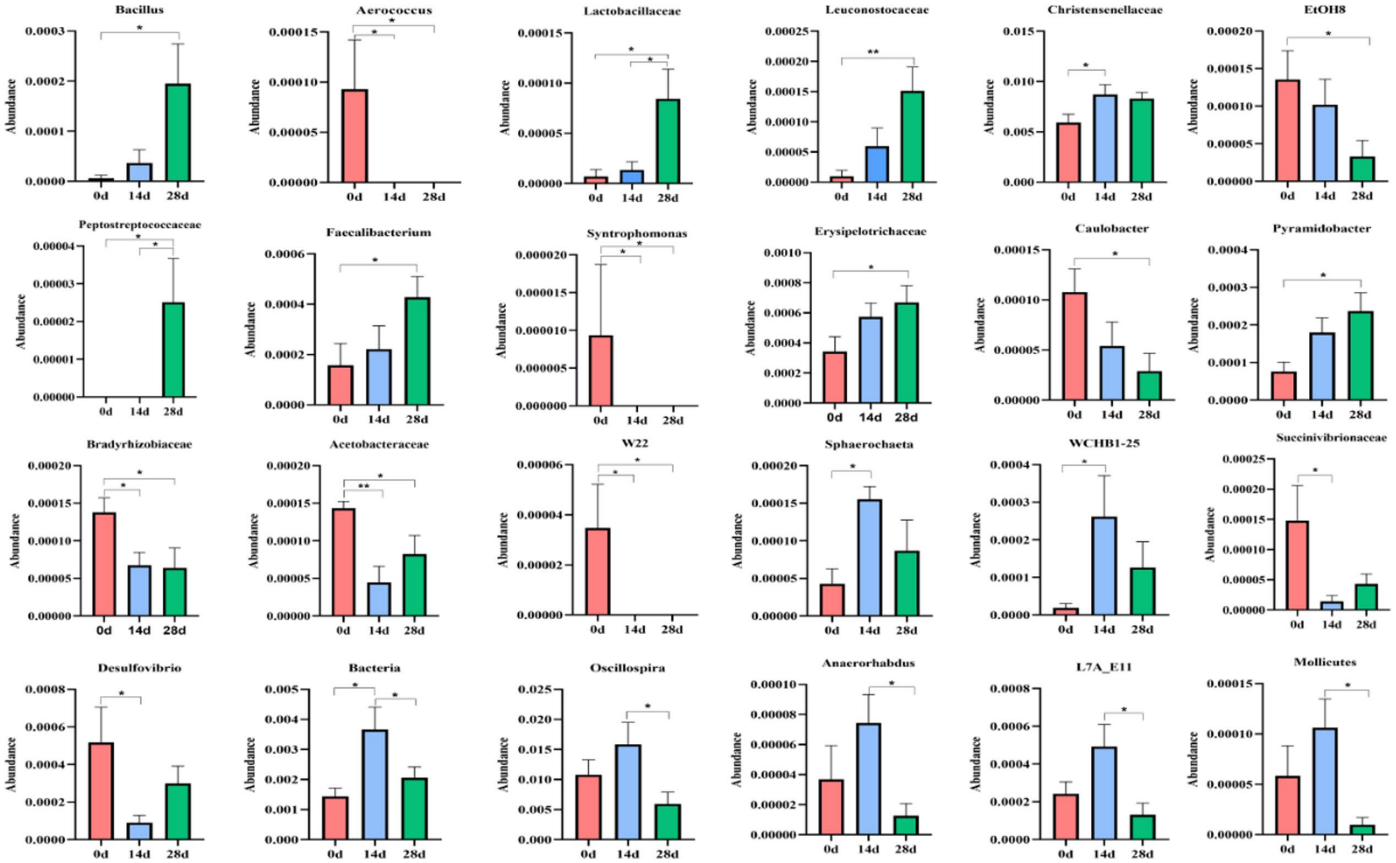
Comparing genera differences in horses’ microbiota. Significance is presented as ^∗^*p* < 0.05, ^∗∗^*p* < 0.01. All data were presented as mean ± SD (*n* = 5).

The abundance of *Lactobacillaceae* (*p* < 0.05) and *Peptostreptococcaceae* (*p* < 0.05) on day 14 was significantly lower than on day 28, respectively. *Bacteria* (*p* < 0.05), *Oscillospira* (*p* < 0.05), *Anaerorhabdus* (*p* < 0.05), *L7A-EAA* (*p* < 0.05), and *Mollicutes* (*p* < 0.05) in 14 were conspicuously higher than on day 28, respectively (Figure 9).

### 3.5 The function predicted of horses’ gut microbiota

**Figure 10:**
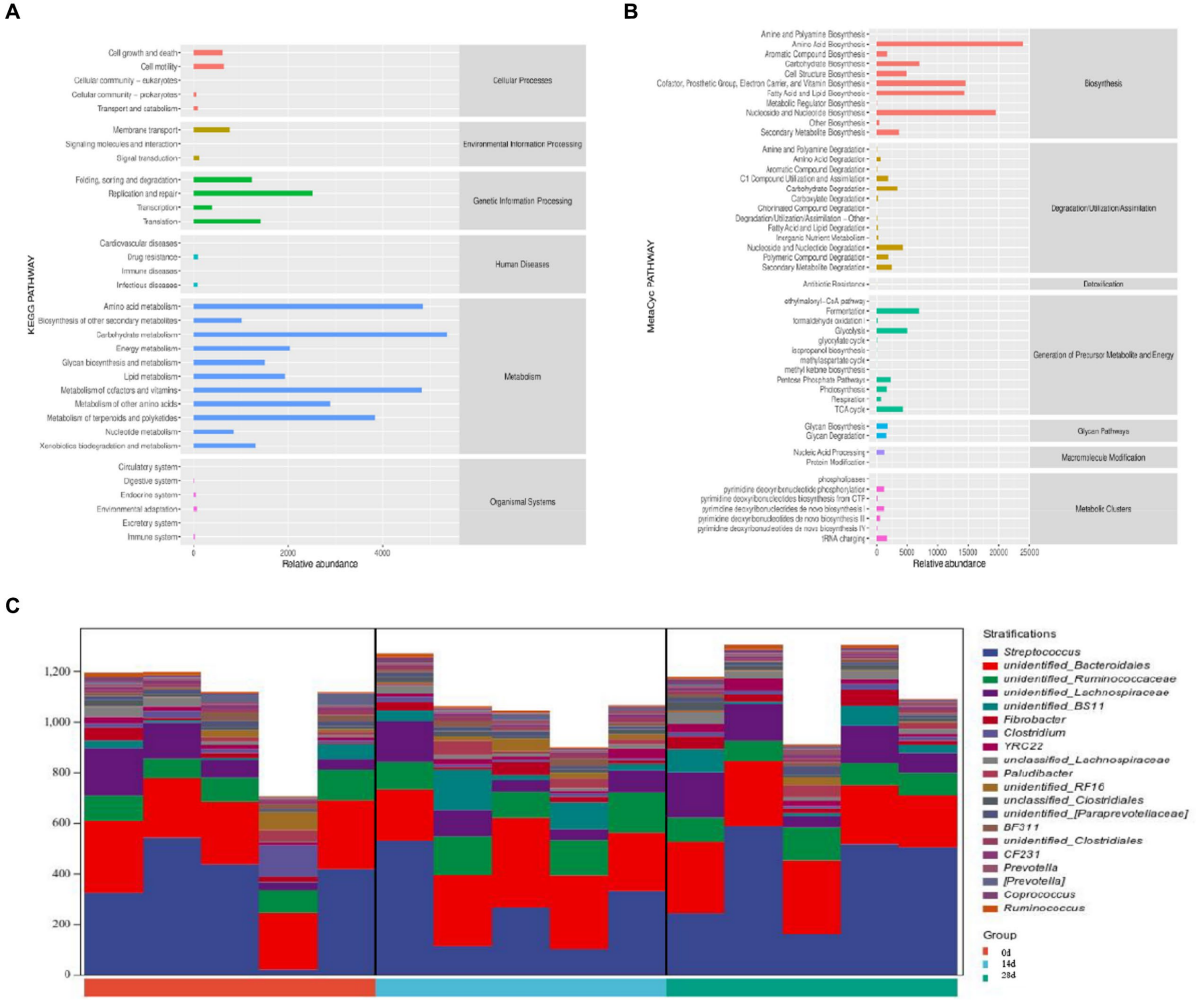
Potential function prediction analysis of horses’ microbiota. **(A)** KEGG, **(B)** MetaCyc, and **(C)** species composition of metabolic pathways.

According to the Kyoto Encyclopedia of Genes and Genomes (KEGG) analysis, the main pathways were connected to metabolism, genetic information processing, and cellular processes. The metabolism pathway included carbohydrate metabolism, amino acid metabolism, and the metabolism of cofactors and vitamins. The genetic information processing pathway includes replication and repair, translation, folding, sorting, and degradation. Cellular processes pathway included cell growth and death cell motility, transport, and catabolism (Figure 10A). According to MetaCyc analysis, the main pathways were related to biosynthesis, generation of precursor metabolites and energy, and degradation/utilization/assimilation. The biosynthesis pathway included amino acid biosynthesis, nucleoside and nucleotide biosynthesis, cofactor, prosthetic group, electron carrier, and vitamin biosynthesis. The generation of precursor metabolites and energy included fermentation, glycolysis, and the TCA cycle. Degradation/utilization/assimilation included nucleoside and nucleotide degradation, carbohydrate degradation, and secondary metabolite degradation (Figure 10B). Pathways are associated with species, and the species composition of metabolic pathways included *Streptococcus, Bacteroidales,* and *Ruminococcaceae* (Figure 10C).

## 4 Discussion

All the examined biochemical profiles and antioxidant enzymes during the experiment were within the range of the horses’ reference values (Stockham, 1995). Therefore, adding HBLS did not affect the health of the horses who were the subject of the study. Additionally, dietary HBLS had a favorable impact on a few of the parameters. Biochemical indices are critical indicators to reflect nutrition, metabolism, stress, and health status. AST, ALT, and ALP are important transaminases that reflect the health status of mitochondria and the cytoplasm of hepatocytes (Rej et al., 1990). CK and LDH are found in skeletal and cardiac muscle and are directly related to cellular energy transport, muscle contraction, and ATP regeneration (Robergs et al., 2004; Wallimann, 2015). In addition, glucose, lipids, and protein are essential nutrients for animals (Chen et al., 2019). Most of the glucose in the body is stored in the liver or muscle, and a small part of the glucose is transported to the tissues and organs with the blood for oxidation and decomposition, providing energy for life activities (TeSlaa et al., 2021). The content of TG and CHO in serum can reflect the development and deposition of adipose tissue (Sakakura et al., 2022). The contents of TP and ALB mainly reflect the body’s nutritional status and protein metabolism level (Suthama et al., 2020). In our experiment, dietary HBLS decreased serum ALT, ALP, and CK activity, and the lowest value was detected on day 28 (*p* < 0.05, *p* < 0.01) (Figure 2). This result is consistent with that of Melchart et al. (2017) and Zhai et al. (2021); it is proven that the Chinese herb compound HBLS has a protective effect on the liver and muscle. The study found that the increase in glucose after the addition of HBLS may be due to the Chinese herb compound being rich in sugars after decomposition into the blood for the body’s energy, so the amount of glucose in the blood will increase.

In recent years, traditional Chinese herbs have been found to have excellent antioxidant activity and have been shown to be effective in scavenging free radicals (Yang et al., 2009). Therefore, using herbs to suppress oxidative stress and reduce free radical damage has attracted widespread attention (Aydin et al., 2016). In the study, we found that supplementing HBLS with diet in horses increased serum T-AOC, SOD, and CAT activity and decreased the MDA level (Figure 3). T-AOC indicates the overall level of various antioxidant substances and antioxidant enzymes and reflects the body’s ability to compensate for external stimuli and the strength of the body’s free radical metabolism (Ahmad et al., 2012; Wang et al., 2017). SOD can catalyze superoxide into oxygen and hydrogen peroxide, thus eliminating the toxicity of the superoxide anion and protecting cells from oxidative damage (Holdom et al., 2000; Lei et al., 2016). CAT could decompose hydrogen peroxide in the body and block the formation of free radicals (Shi et al., 2023). MDA is one of the products of lipid peroxidation, exhibits cytotoxicity and genotoxicity, is an essential indicator of oxidative stress status, and indicates various diseases in the body (Catalan et al., 2010; Mohideen et al., 2023). These results of serum indicators suggested that HBLS could enhance the antioxidant ability of horses.

The microbes or microorganisms in various parts of the gastrointestinal tract are termed “gut microbiota” (Kakakhel et al., 2023b), which is a complex and stable dynamic community consisting of hundreds of aerobic, anaerobic, and alkaline anaerobic bacteria, including fungi, archaea, and bacteria (Frioux et al., 2023). The gut microbiota includes both beneficial and harmful bacteria, so it has a two-way effect on animals: the positive effect of normal flora on the growth and development of animals and their health; there are also pathogenic bacteria on the body caused by negative effects (Caballero-Flores et al., 2023). According to earlier research, traditional Chinese herbs may have impacted the richness and diversity of the gut microbiota (Li et al., 2019; Panyod et al., 2023). The results of alpha and beta diversity analysis revealed that the changes of the values of chao1, observed_species, Faith’PD and Goods coverage (Figure 4); taxa analysis at different levels (Figure 5), classification levels tree diagram, and GraPhlAn evolutionary tree diagram analysis (Figure 6) also pointed out the alteration of dominant bacteria. The above results indicate that supplement of HBLS in horses’ diet significantly improved the structure and diversity of intestinal flora, which may have positive effects on health and digestion.

Further analysis including Venn diagram, heatmap, PCA, OPLS-DA, LEfSe (Figure 7), and random forests (Figure 8) was conducted to reveal different species and their markers in the gut microbiota of horses receiving HBLS. A total of 24 genera were detected in the gut microbiota of horses (Figure 9). The addition of HBLS increased the abundance of *Bacillus, Lactobacillaceae, Leuconostocaceae, Christensenellaceae, Peptostreptococcaceae, Faecalibacterium, Erysipelotrichaceae, Pyramidobacter, Sphaerochaeta, WCHB1-25, Bacteria, Oscillospira,* and *Acetobacteraceae*, while reducing *Aerococcus, EtOH8, Syntrophomonas, Caulobacter, Bradyrhizobiaceae, W22, Succinivibrionaceae,* and *Desulfovibrio*. Although Bacillus are found mainly in the soil, they have also been isolated from the intestinal contents, showing that the bacterium has adapted its physiology to survive under diverse conditions (Hong et al., 2009). Bacillus is an aerobic bacterium that consumes considerable amount of oxygen during its growth. This environment is conducive to the growth of anaerobic bacteria such as Lactobacillus and Bifidobacterium and can inhibit the proliferation of aerobic Enterobacteriaceae; therefore, it can reduce the number of harmful microorganisms in the gut and increase the number of probiotics in the gut, thus reducing the incidence of digestive diseases (Arnaouteli et al., 2021). *Lactobacillaceae* and *Leuconostocaceae* belong to *Lactobacillus*, which propagate in the human and animal intestinal mucosa and are considered probiotics (Dunne et al., 1999; Ouwehand et al., 2002). Intestinal *Lactobacillus* can decompose sugar and produce acid, inhibiting the proliferation of pathogenic and spoilage bacteria. Fuglsang et al. prove that *Lactobacillus* fermentation has been shown to produce powerful bioactive peptides (Fuglsang et al., 2003). Wilk et al.’s results suggest that *Lactobacillus* strains can reduce intestinal inflammation (Wilck et al., 2017). *Christensenellaceae* and *Faecalibacterium* are ubiquitous among humans and other animals and are considered the hallmarks of a healthy gut (Mancabelli et al., 2017). It has been reported that gastrointestinal diseases can cause *Christensenellaceae* and *Faecalibacterium* to decrease (Sokol et al., 2009; Hollister et al., 2020). Adding HBLS to the diet could significantly increase the abundance of *Christensenellaceae* and *Faecalibacterium* in the intestine, which could better protect the intestine and reduce the incidence of intestinal diseases. *Pyramidobacter* genus is an essential member of the *Synergistetes* phylum, which can provide an opportunity for bacterial consortia to protect against the plant toxin fluoroacetic poisoning to protect the gut (Kang et al., 2020). The results of this study are consistent with those of Du et al.; by adding additives to the diet, we can increase the quantity of *Sphaerochaeta* and *Oscillospira* in the intestine to change the abundance of intestinal microflora, thus enhancing the absorption of nutrients in the intestine and improving the body’s resistance (Du et al., 2018; Yang et al., 2021). Additionally, we observed a significant decrease in the abundance of opportunistic pathogenic bacteria, including *Aerococcus* (Jo et al., 2021), *Caulobacter* (Tran et al., 2018), and *Desulfovibrio* (Goldstein et al., 2003).

Although our research has made pronounced progress on the effects of HBLS, there are still some limits to our understanding of HBLS’s long-term effects since the research period is only 28 days. In future research, prolonging the research period is needed to observe HBLS’s long-term effects. Furthermore, we focused on the quantity and diversity of intestinal flora, but the specific functions of flora still needed further investigation to offer a deeper insight into the mechanism of HBLS.

## 5 Conclusion

The integration of HBLS into horses’ diet exhibited notable improvements in antioxidant capacity, liver protection, and alterations in the quantity and diversity of intestinal flora. Our findings underscore the multi-channel, multi-target intervention of the Chinese herbal compound HBLS in horses, providing a robust foundation for future experimental designs. Specifically, the supplementation of HBLS resulted in a significant reduction in ALT, ALP, CK, and MDA levels, coupled with a marked increase in the activity of T-AOC, SOD, and CAT. Notably, continuous feeding of HBLS for 28 days led to further enhancements, particularly in T-AOC CAT levels. It also changed the quantity and diversity of the intestinal flora. This comprehensive evaluation emphasizes the holistic benefits of HBLS, supporting its potential as a valuable dietary supplement for enhancing equine health. More research is needed to investigate the long-term effects and mechanisms of HBLS in horses.

## Data availability statement

The datasets presented in this study can be found in online repositories. The names of the repository/repositories and accession number(s) can be found below: NCBI – PRJNA1033848.

## Ethics statement

The animal study was approved by the Animal Welfare and Ethics Committee of Nanjing Agricultural University. The study was conducted in accordance with the local legislation and institutional requirements.

## Author contributions

JD: Writing – original draft, Software, Methodology. BG: Writing – review & editing. JM: Data curation, Formal analysis, Writing – review & editing. MH: Investigation, Writing – review & editing. WW: Visualization, Writing – review & editing. JL: Supervision, Project administration, Writing – review & editing.
